# Efficacy and safety of enhanced recovery after surgery protocol on minimally invasive bariatric surgery: a meta-analysis

**DOI:** 10.1097/JS9.0000000000000372

**Published:** 2023-03-31

**Authors:** Benjian Gao, Jianfei Chen, Yongfa Liu, Shuai Hu, Rui Wang, Fangyi Peng, Chen Fang, Yu Gan, Song Su, Yunwei Han, Xiaoli Yang, Bo Li

**Affiliations:** a Department of General Surgery (Hepatopancreatobiliary Surgery; b Department of Oncology, The Affiliated Hospital of Southwest Medical University; c Academician (Expert) Workstation of Sichuan Province, Metabolic Hepatobiliary and Pancreatic Diseases Key Laboratory of Luzhou City, The Affiliated Hospital of Southwest Medical University, Luzhou, Sichuan Province, China

**Keywords:** bariatric surgery, enhanced recovery after surgery, meta-analysis, minimally invasive surgery, outcomes

## Abstract

Enhanced recovery after surgery (ERAS), a multidisciplinary and multimodal perioperative care protocol, has been widely used in several surgical fields. However, the effect of this care protocol on patients receiving minimally invasive bariatric surgery remains unclear. This meta-analysis compared the clinical outcomes of the ERAS protocol and standard care (SC) in patients who underwent minimally invasive bariatric surgery.

**Material and methods:**

PubMed, Web of Science, Cochrane Library, and Embase databases were systematically searched to identify literature reporting the effects of the ERAS protocol on clinical outcomes in patients undergoing minimally invasive bariatric surgery. All the articles published until 01 October 2022, were searched, followed by data extraction of the included literature and independent quality assessment. Then, pooled mean difference (MD) and odds ratio with a 95% CI were calculated by either a random-effects or fixed-effects model.

**Results:**

Overall, 21 studies involving 10 764 patients were included in the final analysis. With the ERAS protocol, the length of hospitalization (MD: −1.02, 95% CI: −1.41 to −0.64, *P*<0.00001), hospitalization costs (MD: −678.50, 95% CI: −1196.39 to −160.60, *P*=0.01), and the incidence of 30-day readmission (odds ratio =0.78, 95% CI: 0.63–0.97, *P*=0.02) were significantly reduced. The incidences of overall complications, major complications (Clavien–Dindo grade ≥3), postoperative nausea and vomiting, intra-abdominal bleeding, anastomotic leak, incisional infection, reoperation, and mortality did not differ significantly between the ERAS and SC groups.

**Conclusions:**

The current meta-analysis indicated that the ERAS protocol could be safely and feasibly implemented in the perioperative management of patients receiving minimally invasive bariatric surgery. Compared with SC, this protocol leads to significantly shorter hospitalization lengths, lower 30-day readmission rate, and hospitalization costs. However, no differences were observed in postoperative complications and mortality.

## Introduction

Highlights

- Enhanced recovery after surgery (ERAS) is a multimodal and multidisciplinary perioperative care protocol and has been widely implemented in several surgical fields.
- The effect of the ERAS protocol on patients receiving minimally invasive bariatric surgery remains unclear.
- This is the first meta-analysis of the ERAS protocol versus standard care in patients undergoing minimally invasive bariatric surgery, and the results indicate that this protocol is feasible and safe.

Obesity, a global epidemic, is closely associated with many chronic diseases, such as type 2 diabetes mellitus, dyslipidemia, hypertension, and obstructive sleep apnea, posing a severe threat to public health^1^. Bariatric surgery is considered a conventional treatment for morbid obesity, improving obesity-related comorbidities and, weight loss outcomes better than intensive medical therapy^2^. Therefore, over 1.6 million bariatric surgeries have been performed globally in the past decade. Of them, sleeve gastrectomy (SG) and Roux-en-Y gastric bypass (RYGB) are the two most standard and prevalent surgical approaches, accounting for greater than 80% of all procedures^3^,^4^. Minimally invasive technology offers the advantages of reduced intraoperative bleeding, reduced postoperative complications, and a shortened hospital stay, thus making bariatric surgery more popular^5^,^6^. Nevertheless, the incidence of serious adverse events (such as deep-incisional surgical site infection, pulmonary embolism, pneumonia, stroke/cerebral vascular accident, etc.) remained high during minimally invasive SG (3.53%) and RYGB (7.23%) in super-obese patients^7^.

Enhanced recovery after surgery (ERAS) was first described as an interdisciplinary, standardized, and multimodal care protocol in the late 1990s. This protocol minimizes the surgical stress response and improves postoperative outcomes through perioperative interventions^8^. Compared with conventional perioperative care, the ERAS protocol significantly improves overall morbidity, shortens hospital stay, and reduces hospitalization costs in patients enduring most surgeries, such as gastrointestinal surgery, hepatobiliary surgery, and cardiothoracic surgery^9^–^11^. Patients with morbid obesity commonly experience respiratory, metabolic, and circulatory problems, significantly increasing the difficulty of perioperative care. Simultaneously, these problems challenge the feasibility of implementing the ERAS protocol in the bariatric procedure^12^. In 2016, the ERAS Society reported the first version of evidence-based guidelines for perioperative management in bariatric surgery, updated in 2021. However, many recommendations for this implementation are extrapolated from other surgery types, with relatively low quality of evidence^13^,^14^. Several studies^15^–^35^ have yielded inconsistent outcomes after assessing the effectiveness of this multimodal care protocol in minimally invasive bariatric surgery. Therefore, the safety and usefulness of the ERAS protocol in a bariatric setting are unclear. Thus, a meta-analysis was conducted to evaluate the overall clinical outcomes associated with the ERAS protocol compared with standard care (SC) in patients who underwent a minimally invasive bariatric procedure and to provide a foundation for evidence-based clinical decision-making.

## Material and methods

### Protocol and registration

This systematic review was reported following the Preferred Reporting Items for Systematic Reviews and Meta-Analyses (PRISMA) guidelines^36^ and Assessing the Methodological Quality of Systematic Reviews (AMSTAR) standards^37^. Moreover, the prospective study protocol was registered in PROSPERO (CRD42022308886).

### Search strategy

PubMed, Web of Science, Cochrane Library, and Embase databases were systematically searched from inception to 01 October 2022, to identify all studies reporting the effects of the ERAS protocol on overall clinical outcomes of minimally invasive bariatric surgery. The following search keywords or medical subject heading terms were used in all possible combinations: ‘ERAS,’ ‘ERABS,’ ‘enhanced recovery after surgery,’ ‘enhanced recovery after bariatric surgery,’ ‘fast track,’ ‘laparoscopic,’ ‘robotic,’ ‘minimally invasive,’ ‘bariatric surgery,’ ‘metabolic surgery,’ ‘weight loss surgery,’ ‘diabetes surgery,’ ‘sleeve gastrectomy,’ and ‘Roux-en-Y gastric bypass.’ Furthermore, reference lists of the selected articles were manually examined to identify other relevant articles. Only studies presented in the English language were included in the study.

### Inclusion and exclusion criteria

Three authors (J.C., Y.L., and S.H.) independently determined the eligibility of the articles, and any disagreements were resolved through group discussion. The inclusion criteria were: published randomized controlled trials (RCTs) and retrospective cohort studies; ERAS protocols including at least eight items from the recommended guidelines; a study comparing the influence of the ERAS protocol with that of SC on surgical outcomes in patients after minimally invasive bariatric surgery; and studies whose full texts were available. The latest and most comprehensive data were included if the same institution replicated studies. The exclusion criteria were: abstracts, reviews, case reports, and conference reports; studies without a control group; and studies without a clear description of ERAS protocol elements.

### Data extraction and quality assessment

The full-text screening of the eligible studies was independently conducted by two authors (R.W. and F.P.), followed by data extraction based on the predefined criteria. Consistently, disagreements were resolved through a plenum consensus. The extracted data had details on study characteristics (region, study design, sample size in each arm, and follow-up time), patient baseline features [age, sex, BMI, comorbidity, and type of surgery], ERAS protocol elements, and the clinical outcome indicators [such as hospital length of stay, overall complications, major complications, postoperative nausea and vomiting (PONV), anastomotic leak, intra-abdominal bleeding, incisional infection, reoperation, 30-day readmission, hospitalization costs, and mortality]. The Clavien–Dindo classification was used to assess the severity of complications, with major complications being grade ≥3^38^.

Using the Cochrane Risk of Bias, two authors (C.F., Y.G.) assessed the quality of the included RCTs tool based on seven criteria^39^. Besides, the selected nonrandomized comparative studies were evaluated for risk of bias using the Newcastle–Ottawa scale (NOS), and a score of greater than 6 was considered high-quality^40^.

### Statistical analysis

Pooled estimates for continuous and categorical variables were described as mean difference (MD) and odds ratio (OR), respectively, with a 95% CI. Moreover, heterogeneity between trials was assessed by the *χ* ^2^-test and was expressed using the *I* ^2^ index. Significant heterogeneity was indicated when *P* less than 0.1 or *I* ^2^ greater than 50%. In case of significant heterogeneity, pooled data were calculated with the random-effects model. Otherwise, the fixed-effects model was adopted. Sensitivity analyses were conducted to evaluate the stability of results by removing each study in a single turn and determining the pooled effect size. Further subgroup analyses were performed to determine potential sources of significant heterogeneity, if necessary. The potential publication bias of the included studies was evaluated using contour-enhanced funnel plots. Statistical data analysis was performed using the Cochrane Review Manager software (RevMan, version 5.3), with *P* less than 0.05 being statistically significant.

## Results

### Literature search and study characteristics

**Figure 1:**
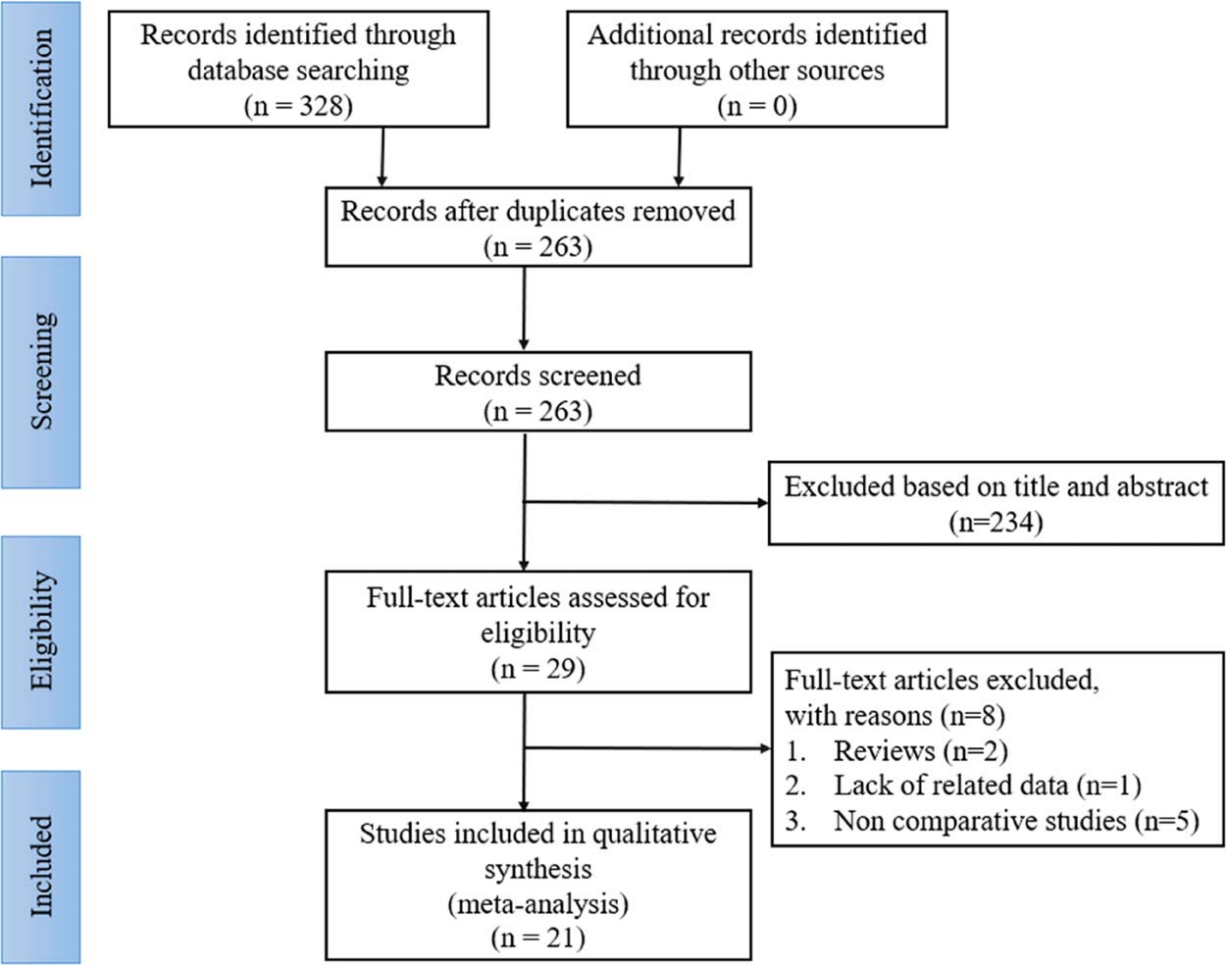
The PRISMA flow diagram of literature selection.

In total, 328 relevant citations were identified using the initial search strategy. Of those, 65 articles were excluded for duplication, and 234 were removed after screening the titles and abstracts. After a detailed full-text review and assessment, eight articles were discarded for not meeting the inclusion criteria. Of the eight articles removed, two were reviews, five had no control group, and one lacked related data. Finally, 21 full-text articles^15^–^35^ involving 10 764 patients were included, of whom 6449 (59.91%) were in the ERAS group and 4315 (40.09%) in the SC group. Figure 1 describes the PRISMA flow diagram of the literature selection process.

Table 1 lists the characteristics of the included studies. All trials comparing the efficacy of the ERAS protocol with that of SC in patients undergoing minimally invasive bariatric surgery were well designed. Of the 21 studies, eight^15^,^17^,^22^,^27^,^28^,^30^,^32^,^35^ were related to laparoscopic SG, three^16^,^21^,^25^ were related to laparoscopic RYGB, and 10^18^–^20^,^23^,^24^,^26^,^29^,^31^,^33^,^34^ were associated with laparoscopic SG + laparoscopic RYGB. Six were RCTs^15^,^17^,^21^,^25^,^28^,^35^, and the remaining 15^16^,^18^–^20^,^22^–^24^,^26^,^27^,^29^–^34^ were retrospective cohort studies. The sample size ranged from 20^17^ to 2064^23^, with the study period ranging between 2013 and 2022. The ERAS and SC groups exhibited no significant difference in sex, age, and BMI.

**Table 1:** Characteristics of the included studies.

References	Region	Design	Type of surgery	Intervention	Sample size	Age (Years)	Sex (M/F)	BMI (Kg/m^2^)	Follow-up (d)	ERAS elements	NOS score
Lemanu 15	New Zealand	RCT	LSG	ERAS	40	43.5±6.9	13/27	46.2±6.1	14	11	–
				SC	38	43.9±6.0	10/28	46.1±6.3			
Dogan 16	Netherlands	RCS	LRYGB	ERAS	75	48.4±8.9	23/52	44.9±5.5	30	11	7
				SC	75	46.2±10.1	23/52	46.8±5.6			
Pimenta 17	Brazil	RCT	LSG	ERAS	10	39.0 (33–45)	1/9	43.5±1.6	60	10	–
				SC	10	32.0 (26–38)	1/9	43.8±1.5			
Simonelli 18	Luxembourg	RCS	LSG+LRYGB	ERAS	103	42.1±11.84	26/77	44.8±5.9	30	12	7
				SC	103	41.5±10.02	29/74	44.3±5.8			
King 19	America	RCS	Mini SG+RYGB	ERAS	715	45 (36–53)	152/563	47 (42–50.8)	30	9	8
				SC	366	46 (36–54)	98/268	48 (42.2–52.8)			
Aleassa 20	America	RCS	LSG+LRYGB	ERAS	134	44 (34–53)	11/123	44 (37–53)	30	10	7
				SC	118	47 (37–55)	15/103	44 (41–50)			
Geubbels 21	Netherlands	RCT	LRYGB	ERAS	110	42.7±10.5	12/98	42.0 (35.2–56.8)	30	13	–
				SC	110	42.6±10.8	16/94	41.4 (35–56)			
Lam 22	America	RCS	LSG	ERAS	130	41.9±10.8	21/109	44.7 (33.7–72.8)	30	11	7
				SC	84	42.7±10.1	23/61	42.4 (30.5–73.7)			
Mannaerts 23	Arab Emirates	RCS	LSG+LRYGB	ERAS	1602	30.41±10.47	572/1030	43.95±5.60	30	11	6
				SC	462	31.55±9.87	147/315	45.82±7.4			
Meunier 24	France	RCS	LSG+LRYGB	ERAS	232	43.07±11	47/185	40.67±6.87	30	17	7
				SC	232	42.98±11	44/188	40.82±6.82			
Ruiz-Tovar 25	Spain	RCT	LRYGB	ERAS	90	45.3±11.7	25/65	44.9±5.5	14	16	–
				SC	90	44.8±10.8	25/65	44.5±4.2			
Trotta 26	Italy	RCS	LSG+LRYGB	ERAS	1019	41.3±11.1	277/742	44.8±67.4	30	12	7
				SC	345	41.5±12	102/243	44.9±67.3			
Jones 27	America	RCS	LSG	ERAS	90	42±11.8	17/73	46.3± 7.3	30	12	8
				SC	570	44±11.2	146/424	45.2±7.2			
Prabhakaran 28	India	RCT	LSG	ERAS	56	36.21±11.31	14/42	42.33±7.01	30	12	–
				SC	56	36.68±9.61	22/34	45.10±8.06			
Ma 29	America	RCS	LSG+LRYGB	ERAS	282	43.9±13.5	N	46.4±8.1	30	10	7
				SC	304	47.0±13.0	N	45.1±6.3			
Yalcin 30	America	RCS	LSG	ERAS	61	17 (16–18)	12/49	51.4 (46.0–60.3)	30	14	8
				SC	51	17 (16–18)	13/38	51.7 (45.6–61.6)			
Gouveia 31	Brazil	RCS	LSG+LRYGB	ERAS	26	39.8±5.3	2/24	42.1±5.3	30	11	7
				SC	56	40.3±9.6	7/49	42.1±4.9			
Sapin 32	America	RCS	LSG	ERAS	1199	40 (15– 74)	244/955	NA	30	12	7
				SC	789	42 (17– 72)	160/629				
Zhou 41	China	RCS	LSG+LRYGB	ERAS	237	32.61±9.60	58/179	38.38±6.78	30	11	7
				SC	198	35.82±10.65	70/128	38.91±7.19			
Diaz-Vico 34	America	RCS	Mini SG+RYGB	ERAS	173	50.2±12.2	47/126	43.6±6.1	90	12	6
				SC	193	48.2±14.3	50/143	44.4±6.1			
Papasavas 35	America	RCT	LSG	ERAS	65	38.0 (30.5, 46.0)	11/54	44.6 (39.8, 47.0)	30	12	–
				SC	65	39.0 (31.0, 50.5)	13/52	42.6 (39.1, 46.6)			

ERAS, enhanced recovery after surgery; F, female; LRYGB, laparoscopic Roux-en-Y gastric bypass; LSG, laparoscopic sleeve gastrectomy; M, male; Mini, minimally invasive (both laparoscopic and robotic surgeries); NA, not available; NOS, Newcastle–Ottawa Scale; RCS, retrospective cohort study; RCT, randomized controlled trial; SC, standard care.

### Quality assessment

**Figure 2:**
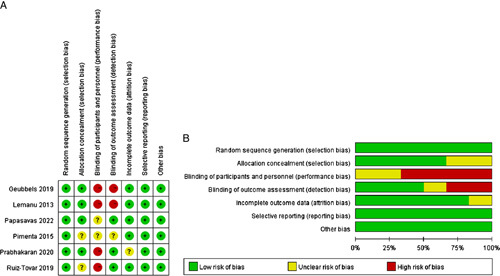
Assessment of risk of bias for each eligible study. (A) Risk of bias summary; (B) Risk of bias graph.

Thirteen of the 15 nonrandomized trials^16^,^18^–^20^,^22^,^24^,^26^,^27^,^29^–^33^ were of high-quality with NOS scores of greater than or equal to 7. The detailed NOS scores of the selected studies are demonstrated in Table 1. Moreover, according to the Cochrane Collaboration risk of bias tool, all included RCTs^15^,^17^,^21^,^25^,^28^,^35^ detailed the methods of random sequence generation. Four studies^15^,^21^,^28^,^35^ concealed allocation using appropriate methods, while the other two^17^,^25^ described nothing about allocation concealment. Three studies^25^,^28^,^35^ blinded outcome assessment, while two^17^,^35^ failed to illustrate participants and personnel blinding. In five studies^15^,^17^,^21^,^25^,^35^, the attrition bias risk was low, while the follow-up data of the remaining trial^28^ were not reported. All studies did not identify selective reporting and other biases^15^,^17^,^21^,^25^,^28^,^35^. The bias assessment of all the RCTs is summarized in Figure 2.

### Meta-analysis results

#### Length of hospitalization

**Figure 3:**
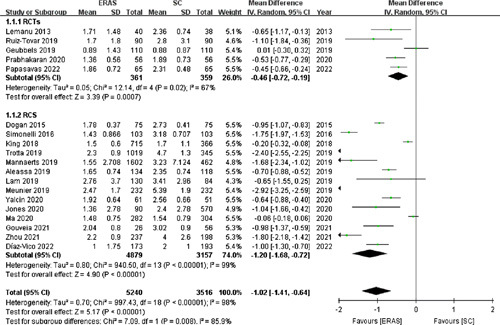
Forest plot of length of hospitalization. RCTs, randomized controlled trials; RCS, retrospective cohort studies.

Nineteen studies^15^,^16^,^18^–^31^,^33^–^35^ (with five RCTs) involving 8756 patients reported the length of hospitalization. Based on the random-effects model, the length of hospitalization was significantly shorter in the ERAS group (MD: −1.02, 95% CI: −1.41 to −0.64, *P*<0.00001; Fig. 3). The weighted mean of the length of hospitalization was 1.77 and 2.87 days in the ERAS and SC groups, respectively.

Similarly, pooled data from the five RCTs^15^,^21^,^25^,^28^,^35^ revealed that the ERAS group had a significantly shorter length of hospitalization than the SC group (MD: −0.46, 95% CI: −0.72 to −0.19, *P*=0.0007).

#### Overall complications

**Figure 4:**
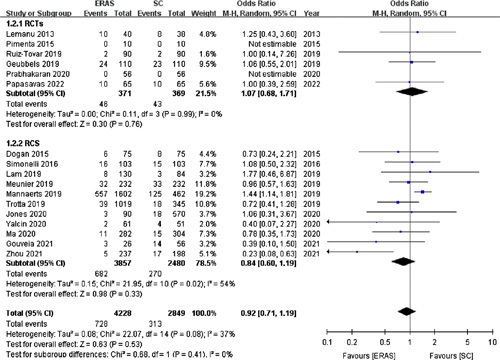
Forest plot of overall complications. RCTs, randomized controlled trials; RCS, retrospective cohort studies.

Seventeen studies^15^–^18^,^21^–^31^,^33^,^35^ (with six RCTs) involving 7077 patients reported the incidence of overall complications. The pooled analysis with a random-effects model indicated that the overall complications had no significant difference between the ERAS (17.22%) and SC (10.99%) groups (OR =0.92; 95% CI: 0.71–1.19; *P*=0.53; Fig. 4).

Consistently, a fixed-effects model for the six RCTs^15^,^17^,^21^,^25^,^28^,^35^ revealed no significant difference in the incidence of overall complications (OR =1.07; 95% CI: 0.68–1.71; *P*=0.76).

#### Major complications

**Figure 5:**
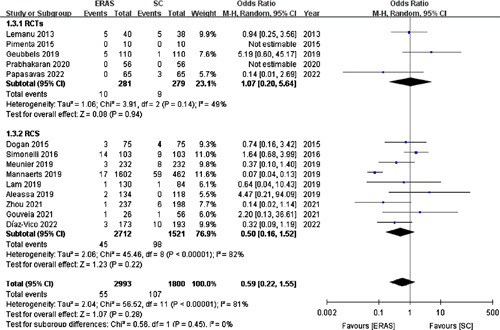
Forest plot of major complications. RCTs, randomized controlled trials; RCS, retrospective cohort studies.

Fourteen studies^15^–^18^,^20^–^24^,^28^,^31^,^33^–^35^ (with five RCTs) involving 4793 patients reported major complications. A random-effects model revealed no significant difference in major complications between the ERAS (1.84%) and SC (5.94%) groups (OR =0.59; 95% CI: 0.22–1.55; *P*=0.28; Fig. 5).

Similarly, a fixed-effects model for the five RCTs^15^,^17^,^21^,^28^,^35^ exhibited no statistical difference in the incidence of major complications (OR =1.08; 95% CI: 0.44–2.69; *P*=0.86).

#### PONV

**Figure 6:**
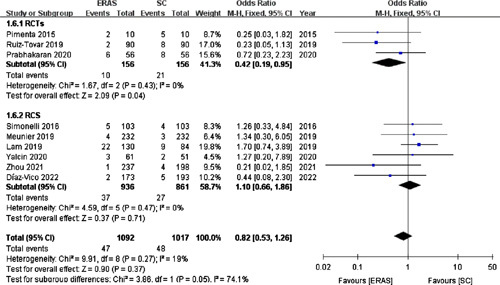
Forest plot of postoperative nausea and vomiting. RCTs, randomized controlled trials; RCS, retrospective cohort studies.

Nine studies^17^,^18^,^22^,^23^,^25^,^28^,^30^,^33^,^34^ (including three RCTs) involving 2109 patients reported the incidence of PONV. The pooled analysis could not reveal any significant difference in the PONV incidence between the ERAS (4.30%) and SC (4.72%) groups (OR =0.82; 95% CI: 0.53–1.26; *P*=0.37; Fig. 6).

In contrast, the pooled data from the three RCTs^17^,^25^,^28^ displayed that the ERAS group patients had a lower PONV rate than those in the SC group (OR =0.42; 95% CI: 0.19–0.95; *P*=0.04).

#### Anastomotic leak

**Figure 7:**
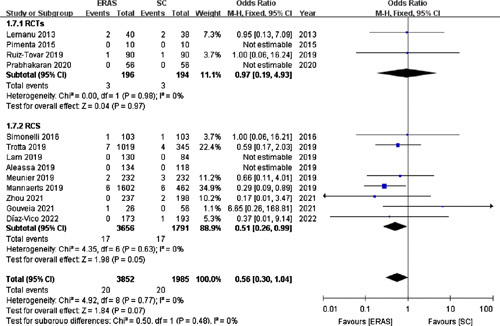
Forest plot of anastomotic leak. RCTs, randomized controlled trials; RCS, retrospective cohort studies.

Thirteen studies^15^,^17^,^18^,^20^,^22^–^26^,^28^,^31^,^33^,^34^ (including four RCTs) with 5837 patients depicted the incidence of anastomotic leak. The entire pooled data revealed no statistical difference in the rate of anastomotic leak between the ERAS (0.52%) and SC (1.01%) groups (OR =0.56; 95% CI: 0.30–1.04; *P*=0.07; Fig. 7).

Similarly, pooled data from the four RCTs^15^,^17^,^25^,^28^ revealed no significant difference in the anastomotic leak rate between the two groups (OR =0.97; 95% CI: 0.19–4.93; *P*=0.97).

#### Intra-abdominal bleeding

**Figure 8:**
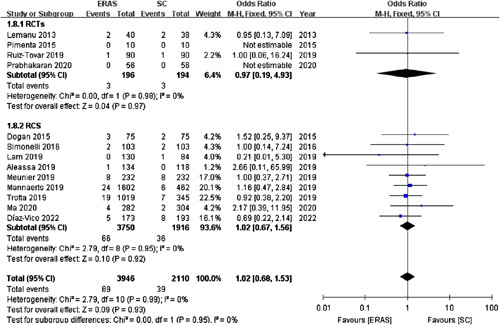
Forest plot of intra-abdominal bleeding. RCTs, randomized controlled trials; RCS, retrospective cohort studies.

Thirteen studies^15^–^18^,^20^,^22^–^26^,^28^,^29^,^34^ (including four RCTs) with 6056 patients reported the incidence of intra-abdominal bleeding. The intra-abdominal bleeding rate did not differ significantly between the ERAS (1.75%) and SC (1.85%) groups (OR =1.02; 95% CI: 0.68–1.53; *P*=0.93; Fig. 8).

Consistently, pooled data from the four RCTs^15^,^17^,^25^,^28^ exhibited that the two groups had a comparable intra-abdominal bleeding rate (OR =0.97; 95% CI: 0.19–4.93; *P*=0.97).

#### Incisional infection

**Figure 9:**
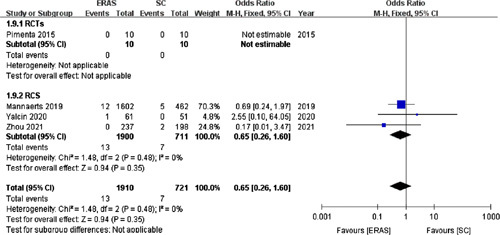
Forest plot of incisional infection. RCTs, randomized controlled trials; RCS, retrospective cohort studies.

Four studies^17^,^23^,^30^,^33^ (with one RCT) involving 2631 patients reported the incidence of incisional infection. No significant difference in the incisional infection rate was observed between the ERAS (0.68%) and SC (0.97%) groups (OR =0.65; 95% CI: 0.26–1.60; *P*=0.35; Fig. 9).

#### Reoperation

**Figure 10:**
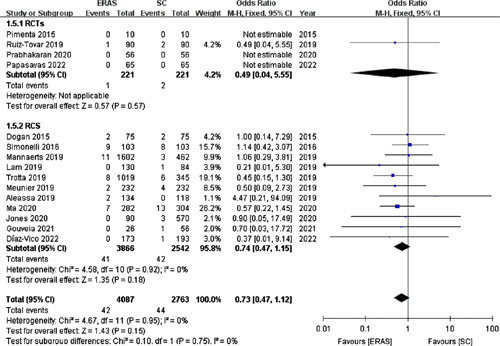
Forest plot of reoperation. RCTs, randomized controlled trials; RCS, retrospective cohort studies.

Fifteen studies^16^–^18^,^20^,^22^–^29^,^31^,^34^,^35^ (including four RCTs) with 6850 patients reported the reoperation rate. No significant difference was observed in the reoperation rate between the ERAS (1.03%) and SC (1.59%) groups (OR =0.73; 95% CI: 0.47–1.12; *P*=0.15; Fig. 10).

Consistently, according to the pooled data from four RCTs^17^,^25^,^28^,^35^, the reoperation rate was comparable (OR =0.49; 95% CI: 0.04–5.55; *P*=0.57).

#### 30-day readmission

**Figure 11:**
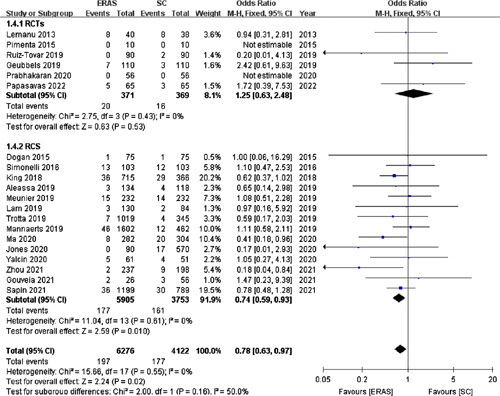
Forest plot of 30-day readmission. RCTs, randomized controlled trials; RCS, retrospective cohort studies.

Twenty studies^15^–^33^,^35^ (including six RCTs) involving 10 398 patients reported the 30-day readmission rate. This rate in the ERAS group was significantly lower (3.14%) than that in the SC group (4.29%) (OR =0.78; 95% CI: 0.63–0.97; *P*=0.02; Fig. 11).

In contrast, pooled data from the 6 RCTs^15^,^17^,^21^,^25^,^28^,^35^ revealed that the two groups had a comparable 30-day readmission rate (OR =1.25; 95% CI: 0.63–2,48; *P*=0.53).

#### Hospitalization costs

**Figure 12:**
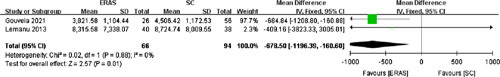
Forest plot of hospitalization costs.

Only two studies^15^,^31^ involving 160 patients reported hospitalization costs. According to the fixed-effects model, the hospitalization costs were significantly lower in the ERAS group (MD: −678.50, 95% CI: −1196.39 to −160.60, *P*=0.01; Fig. 12).

#### Mortality

Seventeen studies^16^–^18^,^20^–^22^,^24^–^31^,^33^–^35^ reported mortality, while only one reported the sudden unexplained death of a patient on postoperative day 19 in the SC group^35^. No significant difference in mortality was observed (OR =0.33; 95% CI: 0.01–8.21; *P*=0.50).

### Sensitivity and subgroup analyses

According to sensitivity analyses, the significant heterogeneity among studies reporting overall and major complications was significantly reduced (*P*=0.55, *I* ^2^=0%; *P*=0.15, *I* ^2^=31%, respectively) by excluding the trial of Mannaerts *et al.* 23. This indicated that heterogeneity might have mainly come from this study. However, the recalculated results revealed no significant change in the outcomes. Furthermore, subgroup analyses were conducted based on surgical types, such as SG, RYGB, and SG+RYGB. The results of the subgroup analyses (Table 2) were similar to those of the primary analyses. However, the hospital stay of RYGB and the 30-day readmission rate of SG and RYGB did not differ significantly between the two groups (MD =−0.65; 95% CI: −1.39 to 0.88; *P*=0.08).

**Table 2:** Subgroup analyses based on surgical types.

Outcomes	SG	RYGB	SG+RYGB
Length of hospitalization (MD; 95% CI)	−0.56 (−0.69 to −0.44), *P*<0.00001	−0.65 (−1.39 to 0.88), *P*=0.08	−1.34 (−2.00 to −0.68), *P*<0.0001
Overall complications (OR; 95% CI)	1.07 (0.63–1.81), *P*=0.80	0.96 (0.56–1.65), *P*=0.89	0.81 (0.53–1.23), *P*=0.32
Major complications (OR; 95% CI)	0.60 (0.21–1.71), *P*=0.34	1.68 (0.25–11.32), *P*=0.59	0.46 (0.12–1.74), *P*=0.25
PONV (OR; 95% CI)	1.08 (0.61–1.93), *P*=0.78	0.23 (0.05–1.13), *P*=0.07	0.74 (0.35–1.58), *P*=0.44
Anastomotic leak (OR; 95% CI)	0.95 (0.13–7.09), *P*=0.96	1.00 (0.06–16.24), *P*=1.00	0.51 (0.26–0.99), *P*=0.05
Intra-abdominal bleeding (OR; 95% CI)	0.59 (0.12–3.05), *P*=0.53	1.34 (0.29–6.12), *P*=0.70	1.04 (0.67–1.61), *P*=0.88
Incisional infection (OR; 95% CI)	2.55 (0.10–64.05), *P*=0.57	–	0.55 (0.21–1.42), *P*=0.22
Reoperation (OR; 95% CI)	0.45 (0.05–4.13), *P*=0.48	0.75 (0.16–3.39), *P*=0.70	0.74 (0.46–1.19), *P*=0.21
30-day readmission (OR; 95% CI)	0.82 (0.56–1.21), *P*=0.33	1.32 (0.47–3.71), *P*=0.60	0.73 (0.56–0.96), *P*=0.02

MD, mean difference; OR, odds ratio; PONV, postoperative nausea and vomiting; RYGB, Roux-en-Y gastric bypass; SG, sleeve gastrectomy.

### Publication bias

**Figure 13:**
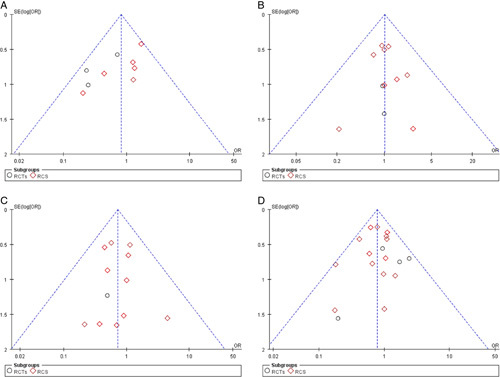
Funnel plot for publication bias in included studies. (A) Postoperative nausea and vomiting; (B) Intra-abdominal bleeding; (C) reoperation; (D) 30-day readmission. RCTs, randomized controlled trials; RCS, retrospective cohort studies.

Funnel plots helped determine the potential publication bias. No significant publication bias was observed through a visual indication of the funnel plots for PONV, intra-abdominal bleeding, reoperation, and 30-day readmission (Fig. 13).

## Discussion

Minimally invasive technology and the ERAS protocol are the two major approaches that could facilitate surgeons in reducing the surgical stress response and accelerating postoperative functional recovery. Since Kehlet first proposed ERAS in patients receiving major surgery, it has been translated into many surgical fields. Moreover, the corresponding evidence-based guidelines for perioperative care have been established to manage patients undergoing surgery^42^,^43^. However, the ERAS protocol was implemented relatively late in bariatric surgery. Several meta-analyses have concluded that the ERAS protocol significantly decreases the length of hospitalization in patients undergoing bariatric surgery^41^,^44^,^45^. Nonetheless, these studies failed to clarify the importance of ERAS in minimally invasive bariatric surgery, even though this surgical approach is strongly recommended^14^. This is the first meta-analysis comparing the ERAS program with SC in patients undergoing minimally invasive bariatric surgery, wherein more recently published studies have been included.

Our results revealed that the ERAS protocol, compared with the SC, significantly decreased the length of hospitalization. The ERAS interventions are carried out throughout the perioperative period to ensure postoperative functional recovery and early discharge. Preoperative education and counseling are strongly recommended as essential ERAS protocol components to ensure realistic expectations and reduce anxiety. All the included studies adopted these recommendations, leading to a shorter hospital stay^14^,^46^. Lam *et al.* 22 reported that the ERAS protocol successfully helped discharge 83.1% of patients on the first postoperative day without any increase in complications and readmissions. Furthermore, some medical centers explored that ERAS-based bariatric surgery could be performed as an outpatient procedure, and same-day discharge seemed feasible and safe in the selected patients^47^,^48^. High-quality evidence is available regarding the advantage of the ERAS protocol in reducing the length of hospitalization in bariatric surgery, as further confirmed by our results in patients undergoing minimally invasive approaches.

Regarding perioperative safety, the ERAS and SC groups had comparable incidences of postoperative complications, including overall complications, major complications, PONV, anastomotic leak, intra-abdominal bleeding, incisional infection, and reoperation. In the stratified analysis, pooled data from three RCTs^17^,^25^,^28^ revealed that the ERAS group had a significantly lower PONV incidence than the SC group. Morbid obesity, gastric surgery, and gastric volume reduction are all risk factors for PONV. Therefore, multimodal PONV prophylaxis should be used in all patients before bariatric surgery based on the ERAS guidelines^14^,^49^. In all the included studies, antiseptic agents, opioid-free total intravenous anesthesia, or multimodal anesthesia were explicitly used as precautionary measures for PONV in the ERAS group, which may have contributed to the lower incidence. Moreover, PONV is the leading cause of delayed discharge and readmission after bariatric surgery^50^,^51^. The 30-day readmission rate was significantly lower in the ERAS group, which differs from that observed in previous studies^41^,^44^,^45^. This may be due to the larger sample size. Besides, PONV and longer hospital stays are predictors of readmission. Another study revealed that patients with a hospital stay of greater than 3 days were 2.57 times more likely to be readmitted than those with a hospital stay of less than or equal to 3 days^52^. In contrast, subgroup analysis from six RCTs revealed that the two groups had no significant difference in the incidence of 30-day readmission. Admittedly, this conclusion should be interpreted cautiously and verified through large-scale RCTs. Our analysis also failed to perceive any statistically significant difference in mortality between the two groups. Venous thromboembolism (VTE) is the primary cause of morbidity and mortality in morbidly obese patients undergoing bariatric surgery^53^. Among all the included studies, only Papasavas *et al.* 35 reported the complications of portal vein thrombosis. Therefore, we did not pool VTE for analysis in our study. The incomplete data could be attributed to the relatively low incidence of VTE in bariatric surgery (<1%)^54^. Moreover, the ERAS protocol implementation is conducive to reducing VTE incidence. In the study of Meunier *et al.* 24, all the patients wore compression stockings during the operation. However, others with a BMI>50 kg/m^2^ or a history of deep venous thrombosis wore pneumatic stockings. Moreover, the combination of mechanical prophylaxis and chemoprophylaxis with unfractionated heparin were also used to prevent the occurrence of VTE as part of the ERAS protocol^27^. Overall, the safety of the ERAS protocol was corroborated in minimally invasive bariatric surgery, which is consistent with most of the included studies.

Previous meta-analyses of the ERAS protocol implementation in bariatric surgery did not determine hospitalization costs. Our study revealed that hospitalization costs were significantly reduced in the ERAS group, indicating the cost-effectiveness of the perioperative interventions. A retrospective study had a significant decline in the median costs by 19.2%, from $11,739.03 to $9482.18, after implementing the ERAS program^55^. Generally, the reduction in length of stay causes a significant decrease in overall hospitalization costs, although the costs of surgical and anesthesia services associated with ERAS will increase accordingly^20^. Stone *et al.* 56 reported that a length of stay reduction by 0.7 days led to a net institutional savings of nearly $400,000 annually.

Postoperative pain can lead to severe suffering in patients and delay functional recovery. Therefore, effective analgesia is a critical component of the ERAS protocol. The ERAS guidelines support using multimodal, opioid-sparing analgesia approaches, including local anesthetics, to advance postoperative recovery for bariatric surgery patients^14^. Lidocaine, dexmedetomidine, ketamine, and nonsteroidal anti-inflammatory drugs possibly have better anti-inflammatory effects and are preferred^57^. In a recent meta‐analysis including eight RCTs^58^, applying the transversus abdominis plane block in laparoscopic bariatric surgery could decrease pain intensity, morphine requirement, and ambulation time, thereby facilitating faster patient recovery. In our meta-analysis, multimodal analgesia protocols were adopted in all the included studies to promote recovery. However, these approaches were very heterogeneous regarding local anesthetic infiltration and drugs. Four studies^20^,^27^,^28^,^32^ reported transversus abdominis plane block application in multimodal analgesia projects, improving efficacy and significantly reducing postoperative opioid use. Moreover, most included studies assessed postoperative pain, but data were not pooled for quantitative analysis due to heterogeneity.

Nonetheless, our meta-analysis has several limitations. First, the number and compliance of ERAS protocol elements varied among the included studies. This could have affected the ERAS efficacy evaluation. Second, heterogeneity could be exacerbated by various baseline characteristics and surgical procedures. Although subgroup analyses were performed, the influence of heterogeneity could not have been eliminated. Finally, only articles published in English were included, leaving out relevant articles in other languages.

## Conclusions

Our study indicated that the ERAS protocol is feasible and safe for patients undergoing minimally invasive bariatric surgery. The protocol significantly reduced the length of hospitalization, hospitalization costs, and 30-day readmission rate without any increase in postoperative complications and mortality. These data can have crucial clinical implications for elevating the quality of evidence, thereby supporting the protocol implementation in patients undergoing minimally invasive bariatric surgery.

## Ethical approval

Ethical approval was not required.

## Sources of funding

This study was supported by the National Natural Science Foundation of China (No. 92059207), the Key Research and Development Project of the Science & Technology Department of Sichuan Province (Nos.22ZDYF1898 and Nos.2021YFS0231).

## Author contribution

B.G., X.Y., B.L.: conception or design of the work; J.C., Y.L., S.H., R.W., F.P., C.F., Y.G.: data collection and quality assessment; B.G., S.S.: analysis and interpretation of data; B.G., J.C., Y.L.: drafting the work; B.G., Y.H., X.Y., B.L.: critical review and final approval.

## Conflicts of interest disclosure

The authors declare that they have no financial conflict of interest with regard to the content of this report.

## Research registration unique identifying number (UIN)

1. Name of the registry: International database of prospectively registered systematic reviews (PROSPERO).
2. Unique Identifying number or registration ID: CRD42022308886.
3. Hyperlink to your specific registration (must be publicly accessible and will be checked): https://www.crd.york.ac.uk/prospero/display_record.php?ID=CRD42022308886.

## Guarantor

Benjian Gao and Bo Li.

## Data availability statement

We are sure our research data available, accessible, discoverable, and useable. All data generated or analyzed during this study are included in this published review article.
